# Theoro-Practical Hints

**Published:** 1862-12

**Authors:** 


### Editorial.

### THEORO-PRACTICAL HINTS.

There is nothing in this world more interesting than an unsatisfied soul. In such condition it is refreshing to watch its workings. We have acquired a periodical appetite for such refreshment, which usually receives its monthly gratification, by reading "Practical Hints" in "The Dental Cosmos." There an earnest professional soul flounders beyond its depth, *unsatisfied* yet undismayed, happening to touch bottom, and determined to reach the shore where the fountain of knowledge wells its bright waters from the depths of science.

Now it must never be inferred from this that our friend is beyond his depth when his "hints" are "*practical*," but when, in spite of their title, they become *theoretical*, then,—

In the September number of the "Cosmos" we are told that, "in testing the mouths of different patients for many years, with litmus and tumeric paper to determine the character of their fluids, we have never yet discovered an alkaline reaction; on the contrary, always an acid."

This is *practical*, but differs from our own observations. But now for theory:—"Using silver plates prove that there is a vast amount of acid exhaled from the blood through the lungs, differing in character in different individuals, but, nevertheless, destructive to the teeth when it is sufficient in quantity."

Now it would be interesting to know exactly how using silver plates proves all this. We say *proves;* for, of course "prove," in the quotation, is a "print error," and the print errors of the "Cosmos" always contrive to get up a discord among verbs and their nominatives.

All breaths contain carbonic acid. It is soluble in saliva. Many exhale hydrosulphuric acid, which is also soluble in saliva. It, too, is readily oxydized, its oxydation resulting in its decomposition, and the formation of sulphuric acid and water. And, if space would permit we might continue to enumerate, but how does silver plate prove all this?

But we are told, "to correct this, when it becomes destructive to the teeth, systematic treatment may be useful, except in those constitutions which, from their own peculiar character, turn everything taken into the system into acid; such are commonly called acid temperaments."

The mild and the strong are certainly well mixed in that sentence. "*May* be useful!" Judicious systematic treatment *must* lessen the acidity of the secretions in such cases. The laws of chemical combination are *laws*—LAWS OF GOD, too, as really as the commandments are.

But if the patient proves to be one of those "acid temperaments," we must just give up. Feed him on beefsteak and eggs, and he turns them to acquafortis or oil of vitriol; give him bread, and it turns to vinegar; stuff him with lime, inject him with soda, and soak him with potash, and you only pickle him. He's a *sour fellow.*

But, come now, Doctor, that wasn't a *practical* hint you gave us just then. But the reader of the "Cosmos" has only to turn a leaf, and the "hints" there are *practical;* and we are sorry they are not more generally followed.

In the October number of the same periodical, the subject is continued, with practical first and theory afterward. In the theoretical, after telling that decay doubtless results from the presence of acids, he remarks:—"That the acid is always the same kind is not probably true," which is evidence that at least one unsatisfied soul is approaching its haven; for the same writer not long ago discovered as follows:—"Some authors have labored to divide caries into several different kinds, but there is but one kind—the only difference is in the degree of intensity with which it makes its progress, etc."

Now we find recognized not only difference in degree, but difference in producing agents, and consequently more than one kind of caries.

But why is it now inferred that the acid is not always the same kind? It is stated that "we find that nearly all the fruits of the season, especially imported fruits which have become stale, destroy tooth substance if kept for a time in contact with each other, and that many daily articles of diet will do the same.

This sentence is ambiguous; but we will try to understand it. Do these fruits, by contact with *each other*, develop electric currents, and do these currents decompose (by electrolysis), the surrounding fluids, so that acids result from the decomposition? Or, do they rot at the points of contact, and do acids result from this spontaneous decomposition? Or, does the author mean that these fruits "destroy tooth substances if kept for a time in contact with" *it?* Possibly, but scarcely probable, or he would certainly have said so. But it is time for dental teachers to abandon the idea that the free acids contained in articles of diet directly induce dental caries, as the disease in its several varieties manifests itself; and it is hghly gratifying to find this writer finishing his compound sentence thus: "but to suppose that it is imperative to abstain from all those things to insure immunity from dental caries is simply foolish." W.

